# Construction of an Escherichia coli Strain Lacking Fimbriae by Deleting 64 Genes and Its Application for Efficient Production of Poly(3-Hydroxybutyrate) and l-Threonine

**DOI:** 10.1128/AEM.00381-21

**Published:** 2021-05-26

**Authors:** Jun Qiao, Xin Tan, Hongyu Ren, Zheng Wu, Xiaoqing Hu, Xiaoyuan Wang

**Affiliations:** a State Key Laboratory of Food Science and Technology, Jiangnan University, Wuxi, Jiangsu Province, China; b Key Laboratory of Industrial Biotechnology, Ministry of Education, School of Biotechnology, Jiangnan University, Wuxi, Jiangsu Province, China; c International Joint Laboratory on Food Safety, Jiangnan University, Wuxi, Jiangsu Province, China; Kyoto University

**Keywords:** *Escherichia coli*, fimbriae, chaperone-usher operon, polyhydroxyalkanoate, poly-3-hydroxybutyrate, l-threonine, transcriptome

## Abstract

Escherichia coli contains 12 chaperone-usher operons for biosynthesis and assembly of various fimbriae. In this study, each of the 12 operons was deleted in E. coli MG1655, and the resulting 12 deletion mutants all grew better than the wild type, especially in the nutrient-deficient M9 medium. When the plasmid pBHR68 containing the key genes for polyhydroxyalkanoate production was introduced into these 12 mutants, each mutant synthesized more polyhydroxyalkanoate than the wild-type control. These results indicate that the fimbria removal in E. coli benefits cell growth and polyhydroxyalkanoate production. Therefore, all 12 chaperone-usher operons, including 64 genes, were deleted in MG1655, resulting in the fimbria-lacking strain WQM026. WQM026 grew better than MG1655, and no fimbria structures were observed on the surface of WQM026 cells. Transcriptomic analysis showed that in WQM026 cells, the genes related to glucose consumption, glycolysis, flagellar synthesis, and biosynthetic pathways of some key amino acids were upregulated, while the tricarboxylic acid cycle-related genes were downregulated. When pBHR68 was introduced into WQM026, huge amounts of poly-3-hydroxybutyrate were produced; when the plasmid pFW01-thrA*BC-rhtC, containing the key genes for l-threonine biosynthesis and transport, was transferred into WQM026, more l-threonine was synthesized than with the control. These results suggest that this fimbria-lacking E. coli WQM026 is a good host for efficient production of polyhydroxyalkanoate and l-threonine and has the potential to be developed into a valuable chassis microorganism.

**IMPORTANCE** In this study, we investigated the interaction between the biosynthesis and assembly of fimbriae and intracellular metabolic networks in E. coli. We found that eliminating fimbriae could effectively improve the production of polyhydroxyalkanoate and l-threonine in E. coli MG1655. These results contribute to understanding the necessity of fimbriae and the advantages of fimbria removal for industrial microorganisms. The knowledge gathered from this study may be applied to the development of superior chassis microorganisms.

## INTRODUCTION

Bacterial fimbriae are large, long, thin, supramolecular protein appendages that appear on the cell surface. They are responsible for biofilm formation, chemotaxis, adhesion, and DNA transfer across cell membranes (1, 2). Fimbriae are also important components of bacterial biofilm and have multiple adverse effects on industrial fermentation of microorganisms, such as increasing the risk of product deterioration or contamination (3), causing serious infections (4), forming dormant cells to increase antibiotics resistance (5), consuming more cellular energy and substrates (6, 7), blocking nutrients diffusion, and forming biofouling to reduce heat transfer, increase corrosion, and shorten the service life of the fermentation equipment (8–10).

**FIG 1:**
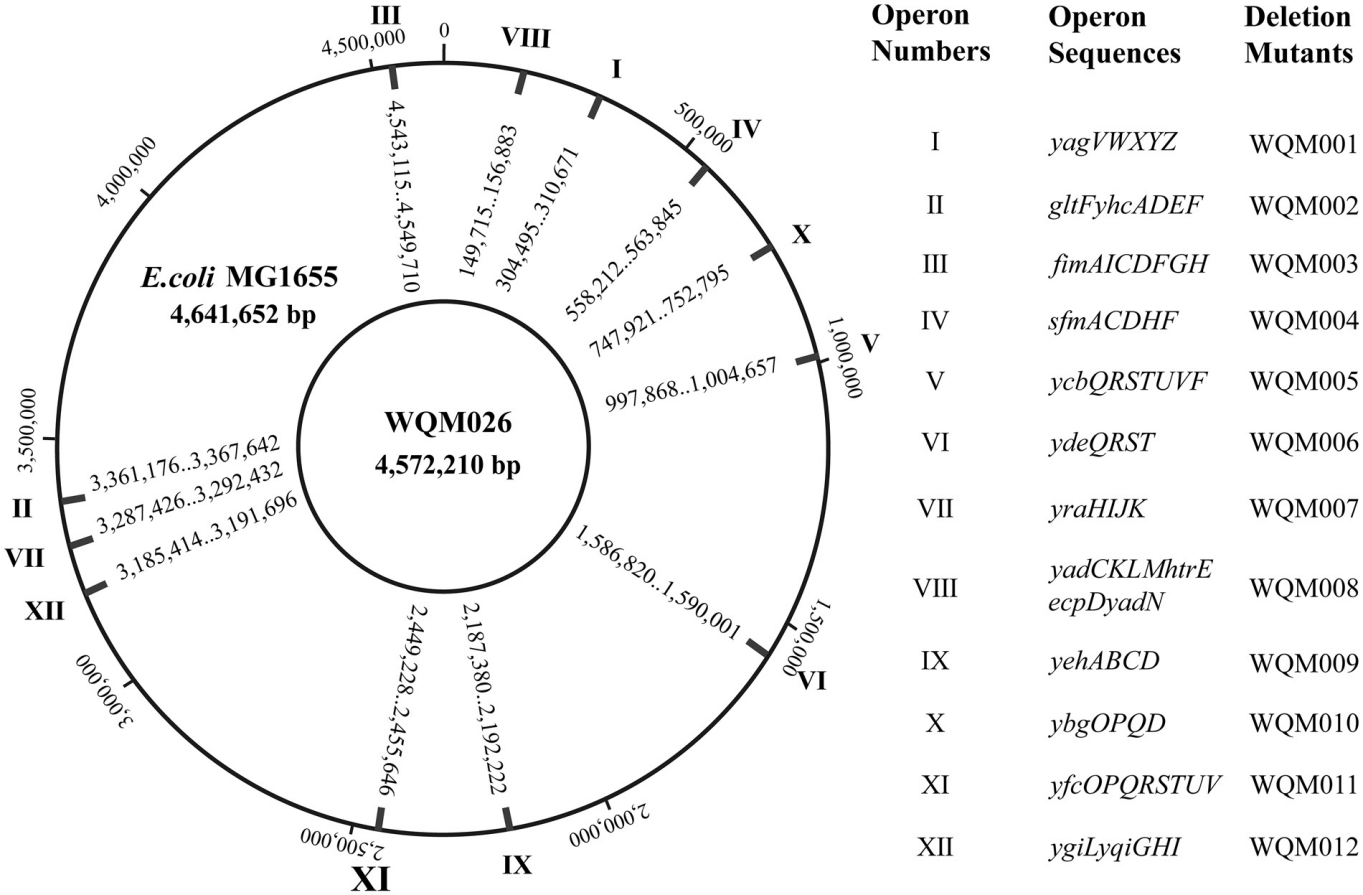
Twelve operons for the synthesis and assembly of CU fimbriae in E. coli MG1655. CU systems are grouped according to the published papers. For more details on prevalence, adhesins, and host receptor molecules, please refer to references 2, 13, 15, and 16.

Bacterial fimbriae are divided into five classes in Gram-negative bacteria according to their different synthetic and secretion systems (11, 12). Among the five classes, the chaperone-usher (CU) fimbriae are the most abundant and have been intensively studied (2, 13, 14). The assembly of CU fimbriae needs the specialized chaperone and usher. The chaperone in periplasm mediates fimbria subunit folding, avoids their polymerization, and guides chaperone fimbria subunit complexes to the pore-forming usher. The usher, located in the outer membrane acting as an assembly platform, facilitates the fimbria subunit protein-chaperone complexes incorporation into the fimbria organelle (2, 11). In Escherichia coli MG1655, there are 12 CU fimbria operons involving 64 genes (Fig. 1). The 12 operons are *yagVWXYZ*, *gltF-yhcADEF*, *fimAICDFGH*, *sfmACDHF*, *ycbQRSTUVF*, *ydeQRST*, *yraHIJK*, *yadCKLM-htrE-ecpD-yadN*, *yehABCD*, *ybgOPQD*, *yfcOPQRSTUV*, and *ygiL-yqiGHI* (15, 16). Fimbriae could cause severe urinary tract infections and improve their recurrent rates (17, 18).

Polyhydroxyalkanoates (PHAs) are synthesized from a variety of hydroxy acyl-coenzyme As (CoAs) as substrates and reserved intracellularly as insoluble spherical inclusions or PHA granules (19–21). It is generally believed that PHAs play an important role in reducing carbon equivalents and storing excess carbon, which could enhance stress resistance of the cells in times of starvation (19, 22, 23). PHAs have biodegradability, biocompatibility, gas barrier, piezoelectric, nonlinear optical activity, and other special properties, based on their functional groups; therefore, they can be used as biodegradable plastics, tissue engineering scaffolds, and many other potential applications (24, 25). At present, the research of PHAs is mainly focused on the modification of their biosynthetic pathway, the development and utilization of inexpensive feedstocks, and the regeneration and modification of PHA (26). l-Threonine is a nutritionally essential amino acid for humans and an important component of protein synthesis. It is widely used in human food, cosmetics, medicine drugs, animal feeds, and health care products (27, 28). The efficient synthesis of l-threonine is mainly focused on metabolic pathway modification, such as fatty acid blockage (29), phosphotransferase system (30), and substrate redistribution (31).

Considering that E. coli fimbriae have pathogenic risks and consume large amounts of substrates and energy in their synthesis and assembly, in this study, all 12 CU operons in E. coli MG1655 have been deleted to investigate their influence on cell growth and production of PHA and l-threonine. The plasmid pBHR68 (32) or pFW01-thrA*BC-rhtC (33) was transferred into these deletion mutants to synthesize PHA or l-threonine, respectively. The results demonstrate that the fimbria-lacking E. coli mutant has the potential to be developed into a chassis bacterium. The strategy used in this study has certain significance in the optimization and transformation of other industrial microorganisms.

## RESULTS

### Removal of any one of the 12 CU pathways in E. coli benefits cell growth and PHA production.

E. coli contains 12 CU pathways to synthesize and assemble fimbriae (Fig. 1). Fimbriae not only contribute to bacterial pathogenicity and persistence (34, 35) but also consume a lot of energy and carbon sources during their synthesis and assembly (36–40). Therefore, removing these fimbriae should improve the biosafety and production efficiency of E. coli.

**FIG 2:**
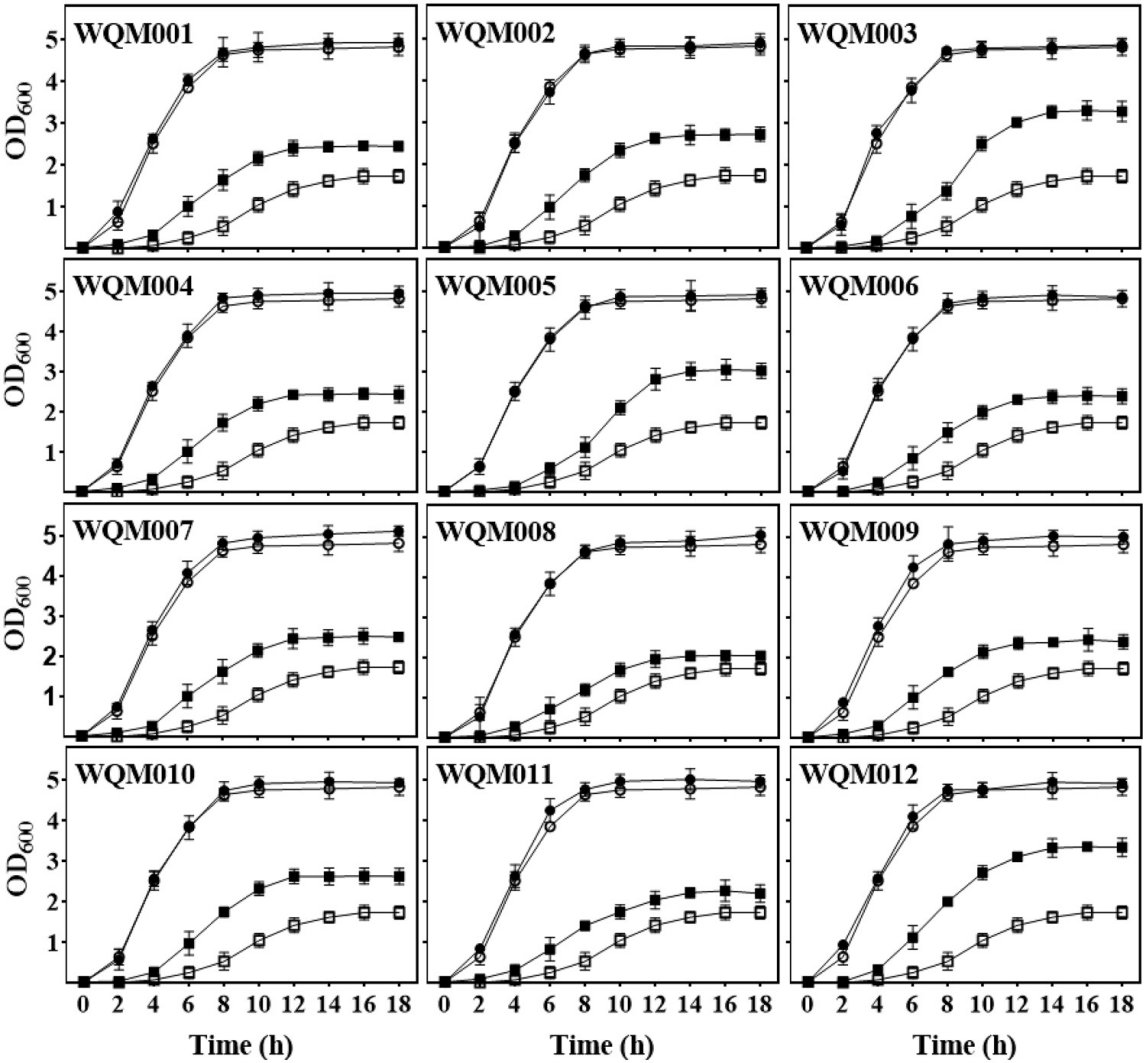
Growth curves of 12 CU fimbria E. coli mutant strains, WQM001, WQM002, WQM003, WQM004, WQM005, WQM006, WQM007, WQM008, WQM009, WQM010, WQM011, and WQM012, in LB and M9 media. Growth curves were drawn by monitoring the OD_600_ value, which represents the bacterial concentration, and the OD_600_ value was measured every 2 h for 18 h. The open symbols represent wild-type E. coli MG1655, and the solid symbols represent the mutant strains. The circle symbols represent the cells grown in LB, and the square symbols represent the cells grown in M9 medium. Three biological replicates were measured for each sample. Error bars represent standard error deviations.

The 12 CU operons were individually removed from the chromosome of E. coli MG1655, resulting in the strains WQM001, WQM002, WQM003, WQM004, WQM005, WQM006, WQM007, WQM008, WQM009, WQM010, WQM011, and WQM012. These strains were cultured in both LB and M9 medium (Fig. 2). In LB medium, all 12 mutant strains showed growth patterns similar to that of control MG1655, suggesting that the removal of any one of the 12 CU pathways in E. coli does not affect or slightly improves cell growth. In M9 medium, however, all 12 mutant strains grew much better than control MG1655. There are no amino acids in M9 medium, and E. coli cells grown in M9 medium have to synthesize all 20 amino acids for protein synthesis (41). The biosynthesis and assembly of fimbriae require a specialized periplasmic chaperone, an outer membrane usher platform, and a large number of transintimal fimbria subunits that would consume a lot of amino acids (2). Therefore, the saved amino acids in the mutants might be used for improving cell growth. This suggests that the removal of fimbriae in E. coli benefits cell growth, especially under nutritional starvation conditions.

**FIG 3:**
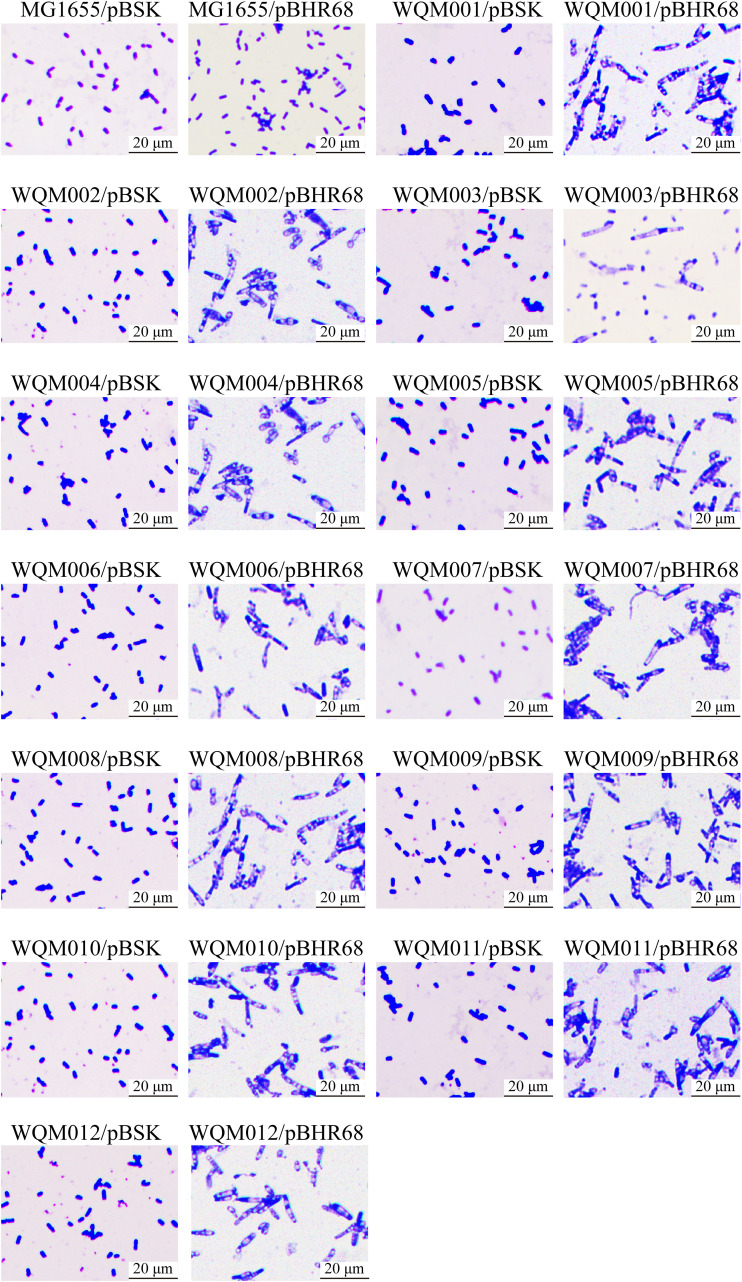
Microscopic observation of E. coli strains MG1655/pBHR68, WQM001/pBHR68, WQM002/pBHR68, WQM003/pBHR68, WQM004/pBHR68, WQM005/pBHR68, WQM006/pBHR68, WQM007/pBHR68, WQM008/pBHR68, WQM009/pBHR68, WQM010/pBHR68, WQM011/pBHR68, and WQM012/pBHR68 grown in M9G medium, together with their vector control strains. Cells were cultured for 24 h, stained by crystal violet, and observed under a light microscope with a 100× oil lens objective. Magnification, ×1,000.

**FIG 4:**
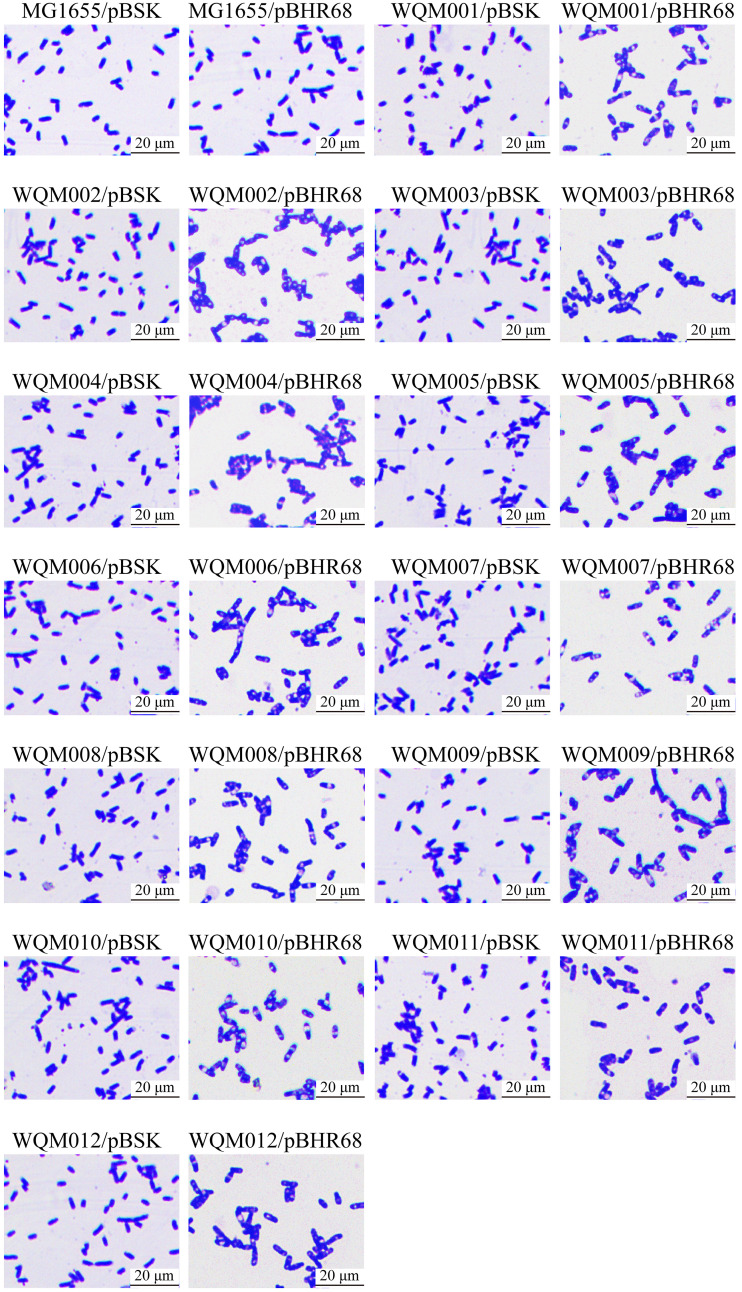
Microscopic observation of E. coli strains MG1655/pBHR68, WQM001/pBHR68, WQM002/pBHR68, WQM003/pBHR68, WQM004/pBHR68, WQM005/pBHR68, WQM006/pBHR68, WQM007/pBHR68, WQM008/pBHR68, WQM009/pBHR68, WQM010/pBHR68, WQM011/pBHR68, and WQM012/pBHR68 grown in LBG medium, together with their vector control strains. Cells were stained by crystal violet and observed with a 100× oil lens objective after 24 h of fermentation. Magnification, ×1,000.

To test the production efficiency of the fimbria mutants, pBHR68, which contains the genes for synthesizing PHA, was introduced into MG1655 and the 12 mutants, resulting in the recombinant strains MG1655/pBHR68, WQM001/pBHR68, WQM002/pBHR68, WQM003/pBHR68, WQM004/pBHR68, WQM005/pBHR68, WQM006/pBHR68, WQM007/pBHR68, WQM008/pBHR68, WQM009/pBHR68, WQM010/pBHR68, WQM011/pBHR68, and WQM012/pBHR68. These recombinant strains were grown in M9G and LBG media, and the cells were observed under the microscope together with their vector controls (Fig. 3 and 4). When grown in M9G medium (Fig. 3), all 13 vector control strains showed similar sizes and did not produce any PHA. MG1655/pBHR68 showed slightly increased size, and only a few cells (at least one cell in Fig. 3) produced PHA. However, WQM001/pBHR68, WQM002/pBHR68, WQM003/pBHR68, WQM004/pBHR68, WQM005/pBHR68, WQM006/pBHR68, WQM007/pBHR68, WQM008/pBHR68, WQM009/pBHR68, WQM010/pBHR68, WQM011/pBHR68, and WQM012/pBHR68 showed significantly enlarged cell size, and almost all cells produced PHA (the white granules inside the cell). Similar patterns of cell size change and PHA production in the 13 recombinant strains and their 13 vector control strains grown in LBG medium were observed (Fig. 4). All 13 vector control strains showed similar sizes and did not produce any PHA. MG1655/pBHR68 showed a size similar to that of its vector control and produced no PHA. The 12 mutants containing pBHR68 showed enlarged cell size and produced PHA with less extent than the corresponding strains grown in M9G medium. This indicates that the removal of any fimbriae could benefit PHA production in E. coli grown in M9G or LBG medium.

### Construction of fimbria-lacking E. coli mutant strain WQM026.

**FIG 5:**
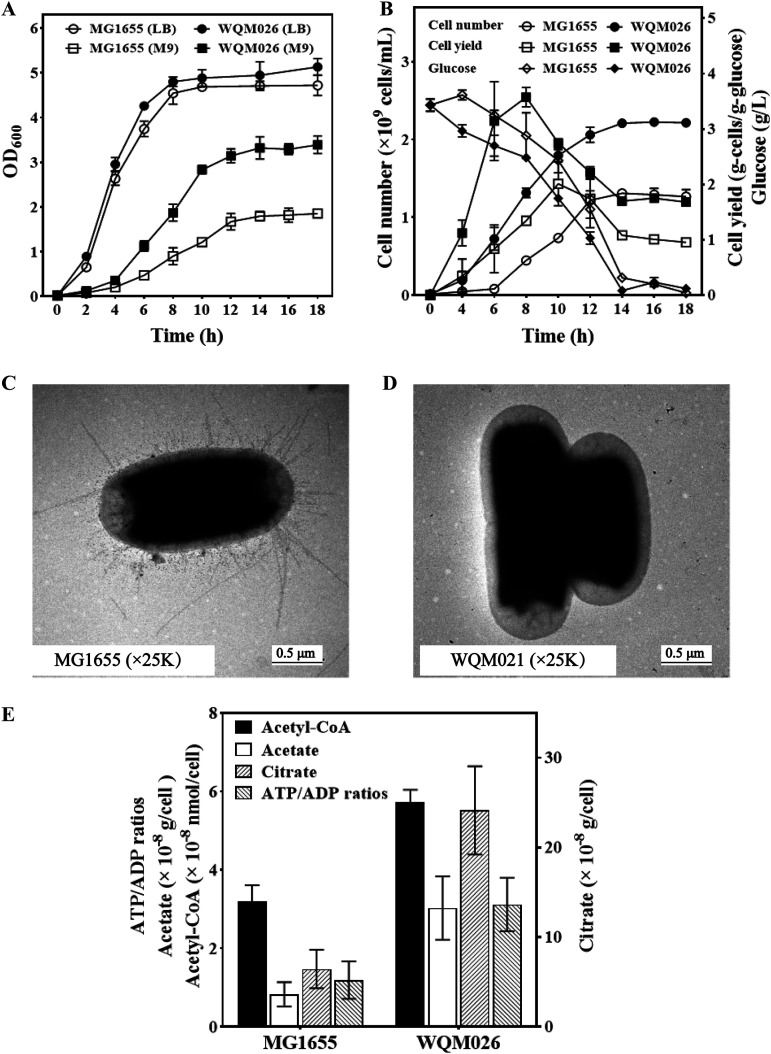
Characterization of the fimbria-lacking E. coli mutant WQM026. (A) Comparison of OD_600_ values of MG1655 and WQM026 grown in LB and M9 medium. (B) Bacterial cell number and glucose consumption of MG1655 and WQM026 during the cultivation in M9 medium. (C) TEM observation of MG1655 cells. (D) TEM observation of WQM026 cells. (E) The intracellular levels of acetyl-CoA, acetate, citrate, and ATP/ADP ratios in MG1655 and WQM026 cells grown in M9 medium. Values in panels A, B, and E represent averages from three independent experiments ± standard deviations.

Since the removal of each fimbria operon could promote cell growth and PHA biosynthesis in E. coli, all 12 CU operons were deleted in the chromosome of E. coli MG1655, resulting in the fimbria-deficient strain WQM026. The strains WQM026 and MG1655 were grown in both LB and M9 media (Fig. 5A). As expected, WQM026 grew slightly better than MG1655 in LB medium and grew much better than MG1655 in M9 medium based on their optical density at 600 nm (OD_600_) (Fig. 5A). Since OD_600_ depends on not only cell concentration but also size and shape of the cells, the numbers of CFU of MG1655 and WQM026 during cultivation in the M9 medium have also been determined (Fig. 5B). After the same time of cultivation, many more cells were produced for the WQM026 strain than for the MG1655 strain, and the WQM026 cell numbers were 1.75-fold higher than MG1655 cell numbers after 14 h of cultivation. The glucose consumption of MG1655 and WQM026 during cultivation in the M9 medium also were determined (Fig. 5B). WQM026 cells consumed glucose much faster than MG1655 cells during cultivation. The yield of WQM026 cells reached the highest (2.54 g-cells/g-glucose) after 8 h of cultivation, while the yield of MG1655 cells was highest (1.43 g-cells/g-glucose) after 10 h of cultivation (Fig. 5B). These results suggest that after the removal of all 12 CU pathways, E. coli cells can use glucose more efficiently for reproduction. MG1655 and WQM026 cells were analyzed by electron microscopy. The surfaces of MG1655 cells were covered with a lot of fimbriae (Fig. 5C), but the surface of WQM026 was smooth and had no signs of fimbriae (Fig. 5D).

To investigate why the absence of fimbriae could produce more PHA in E. coli, concentrations of the intracellular acetate and acetyl-CoA in WQM026 were determined. Acetyl-CoA is the direct precursor of PHA synthesis (19, 21, 42), while acetate is produced from acetyl-CoA (Fig. 5E). The levels of intracellular acetyl-CoA and acetate in WQM026 were significantly higher than that in MG1655. This explains why the removal of fimbria benefits PHA production in E. coli. In addition, the intracellular citrate concentration in WQM026 increased 3.75-fold compared to that of MG1655 (Fig. 5E). The high level of citrate should be derived from the high level of its precursor, acetyl-CoA.

Since the biosynthesis and assembly of various fimbriae need energy (40, 43), the intracellular ATP/ADP ratios in WQM026 and MG1655 were also determined (Fig. 5E). ATP/ADP ratios in WQM026 and MG1655 were 3.12 and 1.19, respectively. This indicates that the removal of fimbriae saves ATP consumption in E. coli.

**FIG 6:**
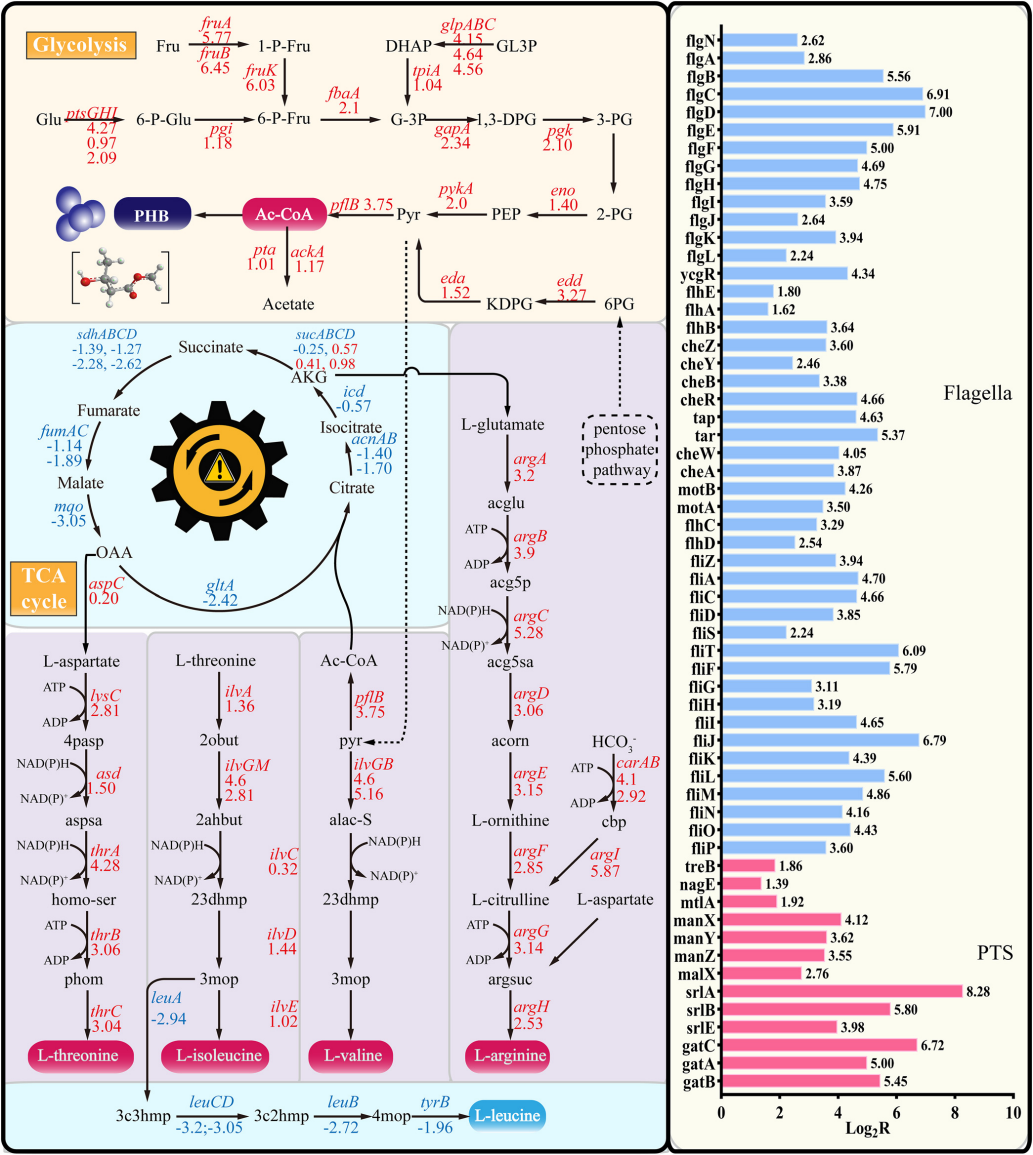
Transcriptional analysis of E. coli WQM026, using MG1655 as a control. Upregulated genes are in red, downregulated genes are in blue. Glu, glucose; 6-P-Glu, glucose-6-phosphate; Fru, fructose; 6-P-Fru, fructose-6-phosphate; 1,6-P-Fru, fructose-1,6-bisphosphate; 1-P-Fru, fructose-1-phosphate; G-3P, glycelaldehyde-3-phosphate; 1,3-DPG, glycerate-1,3-phosphate; 3-PG, 3-phosphoglycerate; 2-PG, 2-phosphoglycerate; PEP, phosphoenol pyruvate; GL3P, glycerate-phosphate; DHAP, dihydroxyacetone phosphate; Pyr, pyruvate; Ac-CoA, acetyl-CoA; acetyl-P, acetyl phosphate; 6PG, d-gluconate 6-phosphate; KDPG, 2-dehydro-3-deoxy-d-gluconate 6-phosphate; AKG, ketoglutaric acid; OAA, oxaloacetic acid; Cit, citrate; 4pasp, 4-phospho-l-aspartate; aspsa, l-aspartate 4-semialdehyde; 2obut, 2-oxobutanoate; homo-ser, l-homoserine; phom, O-phospho-l-homoserine; 2ahbut, (S)-2-aceto-2-hydroxybutanoate; acglu, N-acetyl-l-glutamate; 23dhmp, (R)-2,3-dihydroxy-3-methylpentanoate; 3mop, (S)-3-methyl-2-oxopentanoic acid; acg5p, N-acetyl-l-glutamate 5-phosphate; acg5sa, N-acetyl-l-glutamate 5-semialdehyde; acorn, N-acetylornithine; argsuc, N-(l-arginino)succinate; alac-S, (S)-2-acetolactate; cbp, carbamoyl phosphate; 3c3hmp, (2S)-2-isopropylmalate; 3c2hmp, (2R,3S)-3-isopropylmalate; 3c4mop, (2S)-2-isopropyl-3-oxosuccinate; 4mop, 4-methyl-2-oxopentanoate.

To reveal the effect of fimbria removal on the global metabolic network in WQM026, the transcriptome of WQM026 cells grown in M9 medium at the mid-log phase was analyzed, using MG1655 as a control. Totals of 4,269 and 4,179 transcribed genes were detected in WQM026 and MG1655, respectively. Compared to control MG1655, 2,098 genes were upregulated and 2,081 genes were downregulated in WQM026. Among them, 707 genes (361 genes were upregulated and 346 were downregulated) were significantly regulated (|log_2_*R*| ≥ 1.0, *P* ≤ 0.05). Compared to MG1655, almost all genes in the glycolysis pathway were upregulated in WQM026, while the genes of the tricarboxylic acid (TCA) cycle pathway were downregulated (Fig. 6). This suggests that the carbon sources saved from fimbria synthesis flow mainly to the glycolysis pathway, resulting in increased levels of intracellular acetyl-CoA and acetate in WQM026 (Fig. 5E). The genes related to the biosynthesis of l-aspartate, l-threonine, l-isoleucine, l-valine, and l-arginine were upregulated (Fig. 6). This might be one of the reasons for the better cell growth of WQM026 in M9 medium. Interestingly, the transcriptional levels of genes relevant to l-leucine biosynthesis were downregulated (44–47). Other significantly upregulated genes in WQM026 include all the genes related to flagellar synthesis and assembly and many genes related to the phosphotransferase system (Fig. 6). Except for the genes shown in Fig. 6, other significantly up- or downregulated genes in WQM026 are summarized in Table 1. The 10 upregulated genes were mainly related to transporter and energy regulation, and 7 of the 10 significantly downregulated genes encode rRNA. These data indicate that the removal of fimbriae in E. coli has a global effect on the cells, such as flagella, carbon source utility and rebalance, amino acid biosynthesis, and protein synthesis rebalance.

**TABLE 1:** Top 20 significantly regulated genes, except for the ones shown in Fig. 6

Locus tag	Gene name	log_2_*R*	Annotation
Upregulated
b3547	*yhjX*	7.210522	Putative pyruvate transporter
b0989	*cspH*	7.139243	DNA binding
b4723	*ymcF*	6.620151	Cold shock protein
b2091	*gatD*	6.542079	Galactitol metabolic process
b4665	*ibsC*	6.528019	Toxic peptide
b4245	*pyrB*	6.523107	UMP biosynthesis
b4034	*malE*	6.452172	Maltose ABC transporter protein
b1153	*ymfQ*	6.209994	Uncharacterized protein
b1977	*asnT*	6.209994	tRNA-Asn
b3882	*yihU*	6.209994	NAD binding
Downregulated
b4009	*rrlE*	−15.6541	23S ribosomal RNA
b1544	*ydfK*	−9.85026	Cold shock protein
b1545	*pinQ*	−9.77538	DNA integration
b3971	*rrfB*	−9.73643	5S ribosomal RNA
b3275	*rrlD*	−9.64158	23S ribosomal RNA
b4010	*rrfE*	−9.37997	5S ribosomal RNA
b1375	*ynaE*	−9.37997	Cold shock protein
b3274	*rrfD*	−9.19124	5S ribosomal RNA
b3970	*rrlB*	−9.12677	23S ribosomal RNA
b3272	*rrfF*	−8.79609	5S ribosomal RNA

### The fimbria-lacking E. coli WQM026 is a good host for PHA and l-threonine production.

Compared with the control MG1655, WQM026 has several advantages: better growth and higher accumulation of ATP and acetyl-CoA. Therefore, it is worth exploring its application in the fermentation industry.

**FIG 7:**
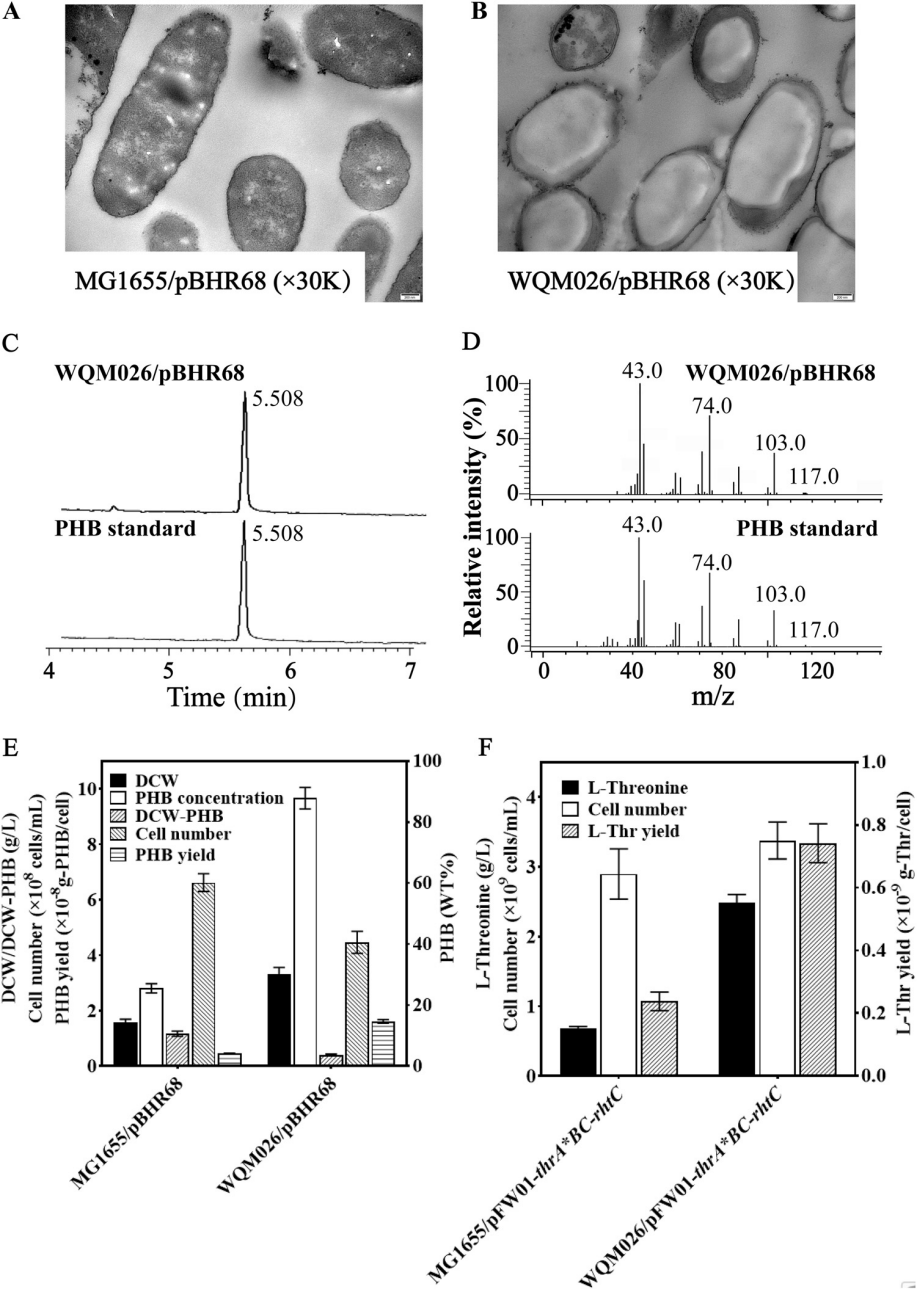
Fimbria-eliminated WQM026 benefits for PHB and l-threonine production. (A) Ultrathin section TEM of MG1655/pBHR68 cells. (B) Ultrathin section TEM of WQM026/pBHR68 cells. (C) GC analysis of PHA. E. coli cells were grown in M9G medium for 48 h, and then PHA granules were extracted, methylated, and analyzed for monomeric composition. (D) GC-MS analysis of PHA structure. (E) Comparison of growth and PHB production in MG1655/pBHR68 and WQM026/pBHR68. (F) l-Threonine production in MG1655/pFW01-thrA*BC-rhtC and WQM026/pFW01-thrA*BC-rhtC strains. Error bars show standard deviations from the means of three independent experiments.

First, the application of WQM026 for PHA production was investigated. The PHA-producing plasmid pBHR68 was introduced into WQM026, resulting in WQM026/pBHR68. The ultrathin-section electron microscopic analysis showed that only very small amounts of PHA were observed in MG1655/pBHR68 cells (Fig. 7A), but WQM026/pBHR68 cells were filled with huge PHB granules (Fig. 7B). This indicates that WQM026 can be used to efficiently synthesize PHA.

PHA can be synthesized by a wide variety of substrates and form intracellular insoluble spherical inclusions or PHA granules (19–21). There are over 90 different PHA monomers (48–51). Introduction of pBHR68 in E. coli strains usually produce poly-3-hydroxybutyrate (PHB) (52, 53), but other types of PHA can also be produced (54, 55). Therefore, gas chromatography-mass spectrometry (GC-MS) was used to determine the type and quantity of PHA in this study. PHA produced by WQM026/pBHR68 was extracted, methyl esterified, and analyzed by GC-MS. In the GC spectrum, only one peak was observed, and its retention time was 5.508 min, which is exactly the same as the retention time of the standard PHB (Fig. 7C). Mass spectrometric analysis of PHA produced by WQM026/pBHR68 also showed the same patterns as the standard PHB (Fig. 7D). This confirms that the PHA produced by WQM026/pBHR68 is PHB. As shown in Fig. 7E, compared with the control MG1655/pBHR68, both the DCW and PHB production of WQM026/pBHR68 significantly increased, reaching 3.31 g/liter and 87.87%, respectively. However, the residual cell weight (DCW-PHB) and bacterial cell numbers of WQM026/pBHR68 were much lower than that of MG1655/pBHR68 (Fig. 7E), resulting in higher cellular PHB production in WQM026/pBHR68. The absence of fimbriae might contribute to the light residual cell weight (56), while the high production of PHB might block cell division and cause reduced cell numbers (57–59). This indicates that this fimbria-lacking WQM026 is a host for efficient PHB production.

Since E. coli has been developed for l-threonine production (29–31, 33, 53), the potential for efficient l-threonine production in WQM026 was also further investigated. The plasmid pFW01-thrA*BC-rhtC, which contains the key genes for l-threonine biosynthesis and transport, was transferred into MG1655 and WQM026, resulting in MG1655/pFW01-thrA*BC-rhtC and WQM026/pFW01-thrA*BC-rhtC, respectively. WQM026/pFW01-thrA*BC-rhtC synthesized more l-threonine than the control strain MG1655/pFW01-thrA*BC-rhtC (Fig. 7F). Different from PHB producing E. coli (Fig. 7E), the bacterial cell numbers of WQM026/pFW01-thrA*BC-rhtC were slightly higher than that of MG1655/pFW01-thrA*BC-rhtC (Fig. 7F). This is understandable, because l-threonine is an important nutrient for E. coli cells. This indicates that fimbria-lacking WQM026 is a host for efficient l-threonine production.

## DISCUSSION

Fimbriae, the nonessential appurtenances located in the extracellular matrix of bacteria, play important roles in protecting bacteria from adverse environmental stresses (60) and in facilitating interactions between bacteria and host cells (61, 62). However, fimbriae show many disadvantages in the fields of medicine, food, and fermentation (3–5, 9, 10). In this study, we constructed a fimbria-lacking E. coli mutant, WQM026, by deleting 64 genes from wild-type E. coli MG1655. WQM026 grew better than MG1655, especially in the nutrient-deficient M9 medium, suggesting that the carbon source and energy saved from the synthesis and assembly of fimbriae were used for the cell growth and biosynthesis of amino acids. This hypothesis is consistent with the findings from the transcriptomic analysis of WQW026. The transcriptional levels of some key genes related to the phosphotransferase system, glycolysis, acetate synthesis, synthesis of various important amino acids (l-aspartate, l-threonine, l-isoleucine, and l-arginine), and flagellar synthesis and assembly were significantly upregulated in WQW026. The results suggest that WQW026 can efficiently consume glucose for better synthesis of some target products. This has been experimentally confirmed by the efficient production of PHA or l-threonine when proper genes were overexpressed in WQW026.

Interestingly, the key genes related to l-leucine biosynthesis were downregulated, while the key genes related to the biosynthesis of l-isoleucine and l-valine were upregulated in WQM026 (Fig. 6). Leucine-responsive regulatory protein (Lrp) binds to the Fim switch to control phase variation of fimbrial expression in E. coli (44), while l-leucine alters the interaction of Lrp with the Fim switch and is needed to promote the phase variation of fimbriae (46). In E. coli WQM026, no fimbriae were synthesized; therefore, the demand for l-leucine decreased, consistent with the downregulation of l-leucine (Fig. 6). In E. coli, the addition of l-leucine to the medium can result in a significant downregulation of the genes relevant to the biosynthesis of l-valine and l-isoleucine (47). This explains the upregulation of l-valine and l-isoleucine in E. coli WQM026 (Fig. 6). In this study, we demonstrate that eliminating fimbriae in E. coli MG1655 can efficiently improve the production of PHA or l-threonine. Considering the pathogenicity of fimbriae, their elimination in E. coli should also make the bacteria safer. A biofilm-deficient E. coli strain, BD123, has been constructed from MG1655 by deleting colanic acid, the curli, and type I pilus encoded by the *wcaL-wza*, *csgG-csgC*, and *fimB-fimH* gene clusters, respectively (8). BD123 existed mainly as planktonic cells and became more intolerant to streptomycin and rifampin than MG1655. The heat shock transformation of BD123 was more efficient than that of MG1655, and the recombinant protein production and secretion of BD123 were more outstanding than those of MG1655. When the core region of lipopolysaccharide in E. coli strain W3110, DH5, or JM109 was removed by deleting *rfaD*, more PHB can be produced (52). Therefore, eliminating macromolecules on the surface of E. coli cells, especially the ones related to pathogenicity (such as fimbriae, biofilm, and lipopolysaccharide), can significantly affect the cell envelope, make the bacteria safer, and save the carbon source and energy consumption. By investigating the necessity of various surface macromolecules in bacteria and the advantages for the bacteria by removing them, such as improving the production of the target products in industrial microorganisms, we can gather knowledge and apply it to the development of superior chassis microorganisms.

## MATERIALS AND METHODS

### Microorganism, culture, and fermentation conditions.

All strains and plasmids used and constructed in this study were listed in Table 2. Strains for plasmid and mutant constructions were cultured at 30°C or 37°C in LB medium (containing 5 g/liter yeast extract, 10 g/liter tryptone, and 10 g/liter NaCl). LB and M9 medium were used to monitor the cell growth. The M9 medium was composed of 4 g/liter glucose, 17.1 g/liter Na_2_HPO_4_·12H_2_O, 4 g/liter KH_2_PO_4_, 3 g/liter NH_4_Cl, 0.5 g/liter NaCl, 0.24 g/liter MgSO_4_, and 0.011 g/liter CaCl_2_. When necessary, 50 mg/liter kanamycin, 50 mg/liter spectinomycin, 100 mg/liter ampicillin, 10 mM arabinose, and 0.5 mM isopropyl-β-d-thiogalactopyranoside (IPTG) were added to the medium.

**TABLE 2:** Bacterial strains and plasmids used in this study

Strain or plasmid	Genotype or description	Source or reference
Strains
JM109	Wild-type E. coli	NEB
MG1655	Wild-type E. coli K-12; F^−^ λ^−^ *rph-1*	CGSC 6300
MG1655/pBSK	MG1655 harboring pBluescript SK−	This study
MG1655/pBHR68	MG1655 harboring pBHR68	This study
WQM001	MG1655△*yagVWXYZ*	This study
WQM002	MG1655△*gltF-yhcADEF*	This study
WQM003	MG1655△*fimAICDFGH*	This study
WQM004	MG1655△*sfmACDHF*	This study
WQM005	MG1655△*ycbQRSTUVF*	This study
WQM006	MG1655△*ydeQRST*	This study
WQM007	MG1655△*yraHIJK*	This study
WQM008	MG1655△*yadCKLM-htrE-ecpD-yadN*	This study
WQM009	MG1655△*yehABCD*	This study
WQM010	MG1655△*ybgOPQD*	This study
WQM011	MG1655△*yfcOPQRSTUV*	This study
WQM012	MG1655△*ygiL-yqiGHI*	This study
WQM001/pBSK	WQM001 harboring pBluescript SK−	This study
WQM002/pBSK	WQM002 harboring pBluescript SK−	This study
WQM003/pBSK	WQM003 harboring pBluescript SK−	This study
WQM004/pBSK	WQM004 harboring pBluescript SK−	This study
WQM005/pBSK	WQM005 harboring pBluescript SK−	This study
WQM006/pBSK	WQM006 harboring pBluescript SK−	This study
WQM007/pBSK	WQM007 harboring pBluescript SK−	This study
WQM008/pBSK	WQM008 harboring pBluescript SK−	This study
WQM009/pBSK	WQM009 harboring pBluescript SK−	This study
WQM010/pBSK	WQM010 harboring pBluescript SK−	This study
WQM011/pBSK	WQM011 harboring pBluescript SK−	This study
WQM012/pBSK	WQM012 harboring pBluescript SK−	This study
WQM001/pBHR68	WQM001 harboring pBHR68	This study
WQM002/pBHR68	WQM002 harboring pBHR68	This study
WQM003/pBHR68	WQM003 harboring pBHR68	This study
WQM004/pBHR68	WQM004 harboring pBHR68	This study
WQM005/pBHR68	WQM005 harboring pBHR68	This study
WQM006/pBHR68	WQM006 harboring pBHR68	This study
WQM007/pBHR68	WQM007 harboring pBHR68	This study
WQM008/pBHR68	WQM008 harboring pBHR68	This study
WQM009/pBHR68	WQM009 harboring pBHR68	This study
WQM010/pBHR68	WQM010 harboring pBHR68	This study
WQM011/pBHR68	WQM011 harboring pBHR68	This study
WQM012/pBHR68	WQM012 harboring pBHR68	This study
WQM026	MG1655△*yagVWXYZ △gltF-yhcADEF △fimAICDFGH △sfmACDHF △ycbQRSTUVF △ydeQRST △yraHIJK △yadCKLM-htrE-ecpD-yadN △yehABCD △ybgOPQD △yfcOPQRSTUV △ygiL-yqiGHI*	This study
WQM026/pBSK	WQM026 harboring pBluescript SK−	This study
WQM026/pBHR68	WQM026 harboring pBHR68	This study
MG1655/pFW01-thrA*BC-rhtC	MG1655 harboring pFW01-thrA*BC-rhtC	This study
WQM026/pFW01-thrA*BC-rhtC	WQM026 harboring pFW01-thrA*BC-rhtC	This study
Plasmids
pBHR68	pBluescript SK− carries *phaCAB* from Ralstonia eutropha	32
pBSK	pBluescript SK−, Amp^r^	Laboratory collection
pCas	*repA101*(Ts) *kan Pcas-cas9 ParaB-Red lacI*^q^*Ptrc-*sgRNA*-pMB1*	63
pFW01-thrA*BC-rhtC	*pFW01* containing thrA*BC and *rhtC*	33
pTargetF	*pMB1 aadA* sgRNA	63
pTargetF01	*pMB1 aadA* sgRNA*-yagVWXYZ*	This study
pTargetF02	*pMB1 aadA* sgRNA*-gltF-yhcADEF*	This study
pTargetF03	*pMB1 aadA* sgRNA*-fimAICDFGH*	This study
pTargetF04	*pMB1 aadA* sgRNA*-sfmACDHF*	This study
pTargetF05	*pMB1 aadA* sgRNA*-ycbQRSTUVF*	This study
pTargetF06	*pMB1 aadA* sgRNA*-ydeQRST*	This study
pTargetF07	*pMB1 aadA* sgRNA*-yraHIJK*	This study
pTargetF08	*pMB1 aadA* sgRNA*-yadCKLM-htrE-ecpD-yadN*	This study
pTargetF09	*pMB1 aadA* sgRNA*-yehABCD*	This study
pTargetF10	*pMB1 aadA* sgRNA*-ybgOPQD*	This study
pTargetF11	*pMB1 aadA* sgRNA*-yfcOPQRSTUV*	This study
pTargetF12	*pMB1 aadA* sgRNA*-ygiL-yqiGHI*	This study

PHA production was fermented in LBG and M9G medium, which were supplemented with 40 g/liter glucose based on LB or M9 medium. Two loops of colonies from the activation plate were incubated into LB seed medium (50 ml medium in a 250-ml flask) and cultured at 37°C for 12 h with 200-rpm shaking. The seeds were then transferred into 50 ml M9G or LBG medium in the 250-ml flask with an initial OD_600_ of 0.1 and fermented for 48 h. The media were supplemented with ampicillin and IPTG to maintain selective pressure and induction of pBHR68 plasmid expression. For the fermentation medium and conditions of l-threonine, please refer to the published literature (31, 33).

### DNA preparation, PCR amplifications, and sequencing.

A Tiangen preparation kit (Tiangen Biotech, Beijing, China) was employed to extract the plasmids and DNA according to the manufacturer's instructions. PCR amplification was performed using 2× Super *Pfu* master mix (CWbio, Beijing, China), and 50-μl PCR contained 25 μl 2× Super *Pfu* master mix, 100 ng template DNA, 1 μl each primer (20 μM), and water. The master cycler from Eppendorf (Hamburg, Germany) was adopted to amplify the target genes: denaturation at 95°C for 5 min, 34 cycles at 95°C for 10 s, 56°C for 30 s, and 72°C for an increased extension period of 4,000 to 6,000 bp/min and a final extension at 72°C for 10 min. Gel extraction kit (Sangon, Shanghai, China) was used to purify DNA from agarose. DNA sequencing was executed by the GENEWIZ Biotechnology Company.

### Construction of various plasmids.

The primers for construction of plasmids and mutants in this study are listed in Table 3. Plasmid pTargetF01, used for deleting the gene cluster *yagV-Z* in the chromosome of E. coli MG1655, was constructed from pTargetF. A linear DNA fragment was produced by inverse PCR from pTargetF using the primers F-single guide RNA (sgRNA)-*yagV-Z* and R-sgRNA. The 50-μl inverse PCR mixture contains 25 μl of 2×Super *Pfu* mater mix, 20 ng of pTargetF, 1 μl of each primer (20 μM), and deionized water. The inverse PCR amplification program was set as denaturation at 95°C for 5 min, 30 cycles of temperature change (at 95°C for 10 s, 56°C for 30 s, and 72°C for 30 s), and a final extension at 72°C for 10 min. The PCR products were purified by gel extraction kit and phosphorylated by T4 polynucleotide kinase at 37°C for 30 min. The phosphorylation reaction mixture contains 2 μl T4 polynucleotide kinase, 2 μl T4 polynucleotide kinase buffer, 10 μl PCR fragments, and 6 μl deionized water. Next, 1 μl T4 DNA ligase was added in the solution of phosphorylation reaction, incubated at 22°C for 4 h, and transformed into E. coli JM109. The resulting plasmid pTargetF01 was sequenced using primers T-*yagV-Z*-F and T-sgRNA-R. The other plasmids, pTargetF02, pTargetF03, pTargetF04, pTargetF05, pTargetF06, pTargetF07, pTargetF08, pTargetF09, pTargetF10, pTargetF11, and pTargetF12, were constructed with the same method with their corresponding primers (Table 3).

**TABLE 3:** Primers used in this study

Primer name	Primer sequence^*a*^ (5′ to 3′)
F1-*yagV-Z*	TGAACTGATTGTGGATATCGAC
R1-*yagV-Z*	CCCTCGACCGATGGATAAGTCTGCATTTCTTCCCGAGTTGAA
F2-*yagV-Z*	TTCAACTCGGGAAGAAATGCAGACTTATCCATCGGTCGAGGG
R2-*yagV-Z*	ACGAAGCCCCGCTATTAT
F-sgRNA-*yagV-Z*	GTGATGATCGATACCGCCAAGTTTTAGAGCTAGAAATAGC
R-sgRNA	ACTAGTATTATACCTAGGACTGAGC
T-*yagV-Z-F*	GTGATGATCGATACCGCCAA
T-sgRNA-R	ACTAGTATTATACCTAGGACTGAGC
F1-*gltF-yhcF*	TTGCCGAAGGTCGTAAGG
R1-*gltF-yhcF*	TTTTGCCCTGTTATGACGGCTGTTGTGAGGTTCTTTTTGA
F2-*gltF-yhcF*	TCAAAAAGAACCTCACAACAGCCGTCATAACAGGGCAAAA
R2-*gltF-yhcF*	AACGGACGTAGGTGGACCT
F-sgRNA-*gltF-yhcF*	GTCATGGCGAGTATACAGTGGTTTTAGAGCTAGAAATAGC
T-*gltF-yhcF-F*	GTCATGGCGAGTATACAGTG
F1-*fimA-H*	GGGGCCAAACTGTCCATA
R1-*fimA-H*	CCCTACTGCTCCTAACGATACCGGCAGAACTGGTTGCTCCTT
F2-*fimA-H*	AAGGAGCAACCAGTTCTGCCGGTATCGTTAGGAGCAGTAGGG
R2-*fimA-H*	GCCATCATTCCTGAAAGCA
F-sgRNA-*fimA-H*	TCGACGCGATACGTCCCTGGGTTTTAGAGCTAGAAATAGC
T-*fimA-H-F*	TCGACGCGATACGTCCCTGG
F1-*sfmA-F*	GTTACGTAGATCGAAGGGG
R1-*sfmA-F*	GCATCTACTTCCTCAGTCGGGTCTGCCGAGTCAGTATTCA
F2-*sfmA-F*	TGAATACTGACTCGGCAGACCCGACTGAGGAAGTAGATGC
R2-*sfmA-F*	TGGCAGAGCGATACAAGC
F-sgRNA-*sfmA-F*	GGGTACGAATATGATTACGAGTTTTAGAGCTAGAAATAGC
T-*sfmA-F-F*	GGGTACGAATATGATTACGA
F1-*ycbQ-F*	TTCGGTGAAGGTCTTGAGT
R1-*ycbQ-F*	GCACAACTCCCACATTACTTGGAATCTTTCGTCGCAGC
F2-*ycbQ-F*	GCTGCGACGAAAGATTCCAAGTAATGTGGGAGTTGTGC
R2-*ycbQ-F*	TCCCGCATAGGCATAGAT
F-sgRNA-*ycbQ-F*	AAATCCCAGACCAAACATGGGTTTTAGAGCTAGAAATAGC
T-*ycbQ-F-F*	AAATCCCAGACCAAACATGG
F1-*ydeQ-T*	ACGGTAGTAAGTGAGATATGGG
R1-*ydeQ-T*	ACCCACTCCCGATGCTTTGGAGGCTTGACGGTGTAA
F2-*ydeQ-T*	TTACACCGTCAAGCCTCCAAAGCATCGGGAGTGGGT
R2-*ydeQ-T*	AGCCTCAAGCTCGTGGTCT
F-sgRNA-*ydeQ-T*	ATGCAGCAAAGGGACAACGGGTTTTAGAGCTAGAAATAGC
T-*ydeQ-T-F*	ATGCAGCAAAGGGACAACGG
F1-*yraH-K*	TCGTTTTCTCACGGCTAA
R1-*yraH-K*	GGTCTACTACCCCAGTTGTACCCATTGATTTCACAAGCGGAT
F2-*yraH-K*	ATCCGCTTGTGAAATCAATGGGTACAACTGGGGTAGTAGACC
R2-*yraH-K*	TGTCGAAGGTCATAAAGCAC
F-sgRNA-*yraH-K*	TCCATCAGAAATCACCCCAGGTTTTAGAGCTAGAAATAGC
T-*yraH-K-F*	TCCATCAGAAATCACCCCAG
F1-*yadC-N*	CTACTCAATCACACACAGCGT
R1-*yadC-N*	AAGAGGCTGTGATGTGCCCATTTTAGCCGTGCTACCT
F2-*yadC-N*	AGGTAGCACGGCTAAAATGGGCACATCACAGCCTCTT
R2-*yadC-N*	AGTGACCACATACAGGAACG
F-sgRNA-*yadC-N*	GCCAGCTATGAGTCACCATGGTTTTAGAGCTAGAAATAGC
T-*yadC-N-F*	GCCAGCTATGAGTCACCATG
F1-*yehA-D*	ATCCTTCGTGGCTGTGTAG
R1-*yehA-D*	CAAGAATAAATCCTACGCCATCAACGAGAGTTTTACACCAG
F2-*yehA-D*	CTGGTGTAAAACTCTCGTTGATGGCGTAGGATTTATTCTTG
R2-*yehA-D*	CAGCCCAGGATGATTCTTA
F-sgRNA-*yehA-D*	TAAATAGAGATAGTAACCCAGTTTTAGAGCTAGAAATAGC
T-*yehA-D-F*	TAAATAGAGATAGTAACCCA
F1-*ybgO-D*	GTCGTGCGAAAGACAAAA
R1-*ybgO-D*	TTTGGGCAGTAGGGTCTTGTTGTCGTGACTTGTCCAAG
F2-*ybgO-D*	CTTGGACAAGTCACGACAACAAGACCCTACTGCCCAAA
R2-*ybgO-D*	CCAATAGAAAGGAGAAGTGATAGC
F-sgRNA-*ybgO-D*	TTAATAATGAAATATCAATGGTTTTAGAGCTAGAAATAGC
T-*ybgO-D-F*	TTAATAATGAAATATCAATG
F1-*yfcO-V*	AAACATCACTATTCGACCCC
R1-*yfcO-V*	TATGTACTGGAACCGGCACGGAACGATAGAACACACTG
F2-*yfcO-V*	CAGTGTGTTCTATCGTTCCGTGCCGGTTCCAGTACATA
R2-*yfcO-V*	GCGGTGAGTGAGGAAAAA
F-sgRNA-*yfcO-V*	CTACGAGTATGACAACCGGGGTTTTAGAGCTAGAAATAGC
T-*yfcO-V-F*	CTACGAGTATGACAACCGGG
F1-*ygiL-yqiI*	ACGCAAGTCCTGTTACGG
R1-*ygiL-yqiI*	CTAAACGGTCCCTCAACAAGCCAGCAACAAGAAGTGAC
F2-*ygiL-yqiI*	GTCACTTCTTGTTGCTGGCTTGTTGAGGGACCGTTTAG
R2-*ygiL-yqiI*	CCTGAAGTGGCTTGATGAC
F-sgRNA-*ygiL-yqiI*	GGGCGAAACAAATAAATACGGTTTTAGAGCTAGAAATAGC
T-*ygiL-yqiI-F*	GGGCGAAACAAATAAATACG

a The N20 sequences in the primers are underlined.

### Construction of various E. coli CU-deficient and PHA-producing strains.

The gene clusters *yagVWXYZ*, *gltF-yhcADEF*, *fimAICDFGH*, *sfmACDHF*, *ycbQRSTUVF*, *ydeQRST*, *yraHIJK*, *yadCKLM-htrE-ecpD-yadN*, *yehABCD*, *ybgOPQD*, and *yfcOPQRSTUV* were knocked out from the MG1655 genome by using the CRISPR-Cas9 method (63), resulting in E. coli mutants WQM001, WQM002, WQM003, WQM004, WQM005, WQM006, WQM007, WQM008, WQM009, WQM010, WQM011, and WQM012, respectively. WQM001 was constructed by deleting the chromosomal *yagVWXYZ*. The upstream and downstream arm fragments were PCR amplified by the primers F1-*yagV-Z*/R1-*yagV-Z* and F2-*yagV-Z*/R2-*yagV-Z*. These two PCR products were recovered with a gel extraction kit and overlapped using the primers F1-*yagV-Z*/R2-*yagV-Z*, resulting in the donor DNA. The purified donor DNA (400 ng) and pTargetF01 (100 ng) were mixed and electroporated into 80 μl MG1655/pCas competent cells. The transformed cells were resuscitated in LB medium for 45 min at 30°C, and then the transformants were spread on an LB agar plate with spectinomycin and kanamycin. After 36 h of incubation at 30°C, F1-*yagV-Z*/R2-*yagV-Z* were used to confirm the correct recombinant strains by colony PCR. The pTargetF01 plasmid then was cured by IPTG overnight induction, and the pCas plasmid was removed by 24 h of culture at 42°C. The mutant WQM001, which lost pTargetF01 and pCas, was used in the following studies. The other 11 mutants, WQM002, WQM003, WQM004, WQM005, WQM006, WQM007, WQM008, WQM009, WQM010, WQM011, and WQM012, were constructed by using the same method. WQM026 was constructed by knocking out all 12 fimbria operons one by one, removing a total of 64 genes. The plasmid pBHR68 (32), carrying *phaCAB* from Ralstonia eutropha and derived from pBluescript SK− (pBSK), was electroporated into E. coli strains MG1655, WQM001, WQM002, WQM003, WQM004, WQM005, WQM006, WQM007, WQM008, WQM009, WQM010, WQM011, WQM012, and WQM026 to investigate the synthesis of PHA, with pBSK as the blank control. Similarly, the plasmid pFW01-thrA*BC-rhtC (33) was electroporated into E. coli MG1655 and WQM026 to explore the synthesis of l-threonine.

### Analytical procedures of the growth curve, amino acid, and organic acid concentration.

Initially, cell growth curves of E. coli strains MG1655, WQM001, WQM002, WQM003, WQM004, WQM005, WQM006, WQM007, WQM008, WQM009, WQM010, WQM011, and WQM012 were measured by monitoring the OD at 600 nm and cell numbers every 2 h. For the final mutant WQM026, the cell growth was evaluated by monitoring the OD at 600 nm and cell numbers every 2 h. Similarly, the cell growth of WQM026/pBHR68 and WQM026/pFW01-thrA*BC-rhtC for PHB and l-threonine production was analyzed by bacterial count with MG1655/pBHR68 and MG1655/pFW01-thrA*BC-rhtC as controls. The serial dilution method was used to enumerate the bacterial count. For testing the intracellular accumulation of organic acids, E. coli cells were cultured in M9 medium until the early mid-log phase (OD_600_, 0.8 to 1.0) and harvested by centrifugation. The cells were washed twice with phosphate-buffered saline (PBS), resuspended in 1 ml PBS, and broken ultrasonically at 300 W for 5 min (2-s sonication, 3-s interval). The supernatants, obtained by centrifugation, were diluted appropriately to analyze the intracellular organic acids. The concentration of intracellular organic acids was analyzed on an e2695-2998 PDA detector high-performance liquid chromatography (HPLC) system (Waters Corporation, Milford, MA) equipped with a 300-mm by 7.8-mm Aminex HPX-87H column (Bio-Rad Laboratories, CA) according to the method reported previously (31). To quantitate the l-threonine production of MG1655/pFW01-thrA*BC-rhtC and WQM026/pFW01-thrA*BC-rhtC, the culture supernatants were obtained by centrifugation at 12,000 rpm for 3 min after 36 h of cultivation. The Agilent 1200 or 1260 series HPLC system, equipped with a Thermo 250-mm by 4.0-mm ODS-2HYPERSIL C_18_ column, was used to quantify amino acids according to the orthophthalaldehyde precolumn derivatization method (64).

### Cell microscopy.

To observe the cell morphology, E. coli cells were grown on solid LB plates for 12 h and imaged with transmission electron microscopy (TEM) (JEM-1200EX; JEOL, Tokyo, Japan) (52). For intracellular PHA granule observation, bacterial suspensions were prepared for light microscopic examination with 1% crystal violet and viewed by bright-field microscopy using an oil immersion lens (magnification power, ×100) (65) after 24 h of PHA fermentation. Meanwhile, E. coli cells were centrifuged at 4,000 rpm for 5 min, washed twice with PBS, and fixed with 2.5% glutaraldehyde solution for a minimum of 72 h. Images were obtained with a G2 spirit transmission electron microscope operated at 100 kV with a Gatan US4000 4kx4k charge-coupled device (FEI Company, Hillsboro, OR) (66).

### Extraction and qualitative and quantitative analyses of PHA.

E. coli cells harboring pBHR68 were cultured in M9G or LBG medium for 48 h at 37°C and centrifuged at 12,000 rpm for 3 min. The PHA extraction with methanol and chloroform was carried out according to previously published methods (53). The extracts were analyzed to reveal the PHA composition and accurate content in the flame electron ionization mode of GC-MS. The GC-MS was a Scion SQ-456-GC (Bruker Daltonic, Billerica, MA) model coupled with a triple quadrupole mass spectrometer and a DB-5MS fused silica capillary column (30 m by 0.25 mm by 0.25 μm). The GC detective process was based on a published methodology (54). Positive electron ionization (EI) was obtained using ionization energy of 70 eV, and mass spectra were programmed by scanning ions from *m/z* 50 to *m/z* 650 at a scan interval of 0.5 s. The PHA production was reported as the percent composition of dry cell weight (DCW).

### Extraction and determination of intracellular ATP/ADP ratios and acetyl-CoA.

E. coli cells were cultured in M9 medium until early mid-log phase (OD_600_, 0.8 to 1.0) and collected for the quantitative determination of intracellular ATP/ADP ratios and acetyl-CoA levels using the ATP assay kit (Solarbio, Beijing, China), ADP assay kit (Solarbio, Beijing, China), and acetyl-CoA assay kit (Solarbio, Beijing, China). Extraction and quantification for intracellular ATP/ADP ratios were carried out according to the ATP and ADP assay kit instructions, and the analysis of ATP/ADP ratios was performed with an Agilent 1260 series HPLC system. The analytical column was a Thermo 250-mm by 4.0-mm ODS-2HYPERSIL C_18_ column. The intracellular acetyl-CoA level was analyzed according to previous publications (52, 53).

### Transcriptome analysis of MG1655 and WQM026.

E. coli MG1655 and WQM026 were cultivated to mid-exponential phase in M9 medium, harvested at 12,000 rpm for 3 min, washed twice by PBS, and frozen quickly in liquid nitrogen. The extraction, construction, and sequencing of RNA libraries were executed by the GENEWIZ Biotechnology Company. The whole-transcriptome analysis proceeded by following the published method (67, 68). The MG1655 genome, as the reference sequence, was used for sequence reading, alignment, and analysis. Differential gene expression was calculated based on their expression levels and *P* values of ≤0.05 by the FIESTA Viewer v.1.0 software (69).

### Data availability.

Raw transcriptome sequence data are available from the NCBI Sequence Read Archive (SRA) under BioProject accession code PRJNA683932.
